# Mapping mitochondrial heteroplasmy in a Leydig tumor by laser capture micro-dissection and cycling temperature capillary electrophoresis

**DOI:** 10.1186/s12907-017-0042-3

**Published:** 2017-04-08

**Authors:** Paulo Refinetti, Christian Arstad, William G. Thilly, Stephan Morgenthaler, Per Olaf Ekstrøm

**Affiliations:** 1 Chair of Applied Statistics, Ecole Polytechnique Federale de Lausanne, Lausanne, Switzerland; 2 Institute for Cancer Research, The Norwegian Radium Hospital, Oslo, Norway; 3 Laboratory in Metakaryotic Biology, Massachusetts Institute of Technology, Cambridge, MA USA

**Keywords:** Capillary electrophoresis, Mitochondrial mutations, Heteroplasmy, Homoplasmy, LCM, Tumor heterogeneity

## Abstract

**Background:**

The growth of tumor cells is accompanied by mutations in nuclear and mitochondrial genomes creating marked genetic heterogeneity. Tumors also contain non-tumor cells of various origins. An observed somatic mitochondrial mutation would have occurred in a founding cell and spread through cell division. Micro-anatomical dissection of a tumor coupled with assays for mitochondrial point mutations permits new insights into this growth process. More generally, the ability to detect and trace, at a histological level, somatic mitochondrial mutations in human tissues and tumors, makes these mutations into markers for lineage tracing.

**Method:**

A tumor was first sampled by a large punch biopsy and scanned for any significant degree of heteroplasmy in a set of sequences containing known mutational hotspots of the mitochondrial genome. A heteroplasmic tumor was sliced at a 12 μm thickness and placed on membranes. Laser capture micro-dissection was used to take 25000 μm^2^ subsamples or spots. After DNA amplification, cycling temperature capillary electrophoresis (CTCE) was used on the laser captured samples to quantify mitochondrial mutant fractions.

**Results:**

Of six testicular tumors studied, one, a Leydig tumor, was discovered to carry a detectable degree of heteroplasmy for two separate point mutations: a C → T mutation at bp 64 and a T → C mutation found at bp 152. From this tumor, 381 spots were sampled with laser capture micro-dissection. The ordered distribution of spots exhibited a wide range of fractions of the mutant sequences from 0 to 100% mutant copies. The two mutations co-distributed in the growing tumor indicating they were present on the same genome copies in the founding cell.

**Conclusion:**

Laser capture microdissection of sliced tumor samples coupled with CTCE-based point mutation assays provides an effective and practical means to obtain maps of mitochondrial mutational heteroplasmy within human tumors.

## Introduction

The mitochondrial genome (mtDNA) is a circular DNA molecule [1, 2] of around 16.5 kb. Multiple copies of mtDNA are found in each mitochondrion and a human cell may have between 100 and 10000 copies [3], depending on cell type. In early studies of somatic mtDNA mutations in tumors, it was suggested that somatic mitochondrial mutations can grow from a low frequency of occurrence (heteroplasmy) to become completely dominant (homoplasmy). Homoplasmy was reported to be found in more than half of human tumors [4–8]. Later studies, however, have found this number to be significantly lower [9, 10]. Hypothetical paths to homoplasmy, however, can be offered as mathematical models based on random drift linked to mtDNA replication during cell division and in the absence of selection [5, 11, 12]. The basic hypothesis is that once a mutation arises in an organogenic stem cell as a single copy of mtDNA, this mutant copy may be retained in a stem cell lineage and by random distribution result in some stem cells to contain a significant fraction of a particular mutant mtDNA copy. If said stem cell becomes the first tumor stem cell then subsequent tumor stem cell divisions would be expected to continue random distribution of mutant copies so that after multiple divisions some descendant copies would contain no mutant copies (wild type homoplasmy) while others would have all copies carrying the mutant sequence (mutant homoplasmy) with most stem cells distributed with intermediate forms of mutant heteroplasmy.

It is generally believed that most tumors are heterogeneous with infiltrations of non-tumor cells [13]. Sampling of only tumor-derived cells is therefore difficult. As non-tumor cells would not carry the mutation present in tumor cells it would not be possible to distinguish between heteroplasmy and homoplasmy in a random tumor sample [14, 15]. Pathological evaluation is often used to locate the tumor tissue. The success of this method depends on the experience of the pathologist and the quality of staining [8, 16–18].

Somatic mtDNA mutations have been used as a marker of clonality in human tissues or tumors [11]. A successful method has been the immuno-histochemical marking of cytochrome oxidase (COX) deficient cells [19–21].

In Taylor et al. [3], selected individual colonic crypts were sequenced and sixty mitochondrial mutations were detected, of which half were homoplasmic. None of the mutations were identical. The probability of independent identical homoplasmic mutations can be assumed to be very low, as is also predicted by the mathematical model [11].

Laser capture microdissection (LCM) was introduced in 1996 [22]. The method uses a laser to cut selected areas of tissue resting on a membrane. The area of interest can be collected by gravity or by adherence and is transferred to post processing vials. LCM has been used to capture spots of tissue for the analysis of proteins, mRNA and DNA [23–26].

This study describes a method to trace somatic mitochondrial mutations through a tumor slice. In a first step, DNA from a large representative piece of tissue is analyzed to detect the presence of mtDNA mutations. If mutations are found, LCM is used to obtain several small spots of the tissue. Cycling capillary temperature electrophoresis (CTCE) [27–30] is used to detect and quantify mutant fractions in each sample. In the course of this study, more than 900 LCM samples were analyzed for the presence of mtDNA mutations.

## Materials and methods

### Tissue samples

Surgical discharges were collected under informed consent at the surgical departments of Bærum sykehus (Vester Viken, Helse sør-øst, Norway) in 2015. Similarly, liver samples were collected as surgical discharges under informed consent at the University Hospital in Bologna (Policlinico Sant’Orsola-Malpighi, Bologna, Italy) as part of a different study.

According to the regional ethics comity (REC), “technical and methodological development work that uses anonymized biological material” does not require approval from REC (https://helseforskning.etikkom.no/ikbViewer/page/reglerogrutiner/soknadsplikt/sokerikkerek?p_dim=34999&_ikbLanguageCode=us).

Six samples of testicular cancer, five germ cell tumors, and one Leydig cell tumor were snap-frozen in liquid nitrogen and stored at −70 °C until DNA extraction was performed. Fresh frozen samples have been shown to have better DNA integrity than formalin fixed, paraffin embedded samples [31]. All samples were anonymized with an arbitrary number.

### DNA extraction

A representative sample of each tissue was obtained by inserting a blunt end 19G 11/2 hypodermic needle (Microlance3, Becton Dickinson, Ireland) through the frozen tissue. DNA was extracted from the core sample by Blood & Cell Culture DNA Mini Kit (Qiagen, Valencia, California USA). DNA was eluted with elution buffer (as recommended by manufacturer) and stored at −20 °C. The DNA from these samples was analyzed for somatic mitochondrial mutations.

### First round PCR

Segments of mtDNA that have previously shown to contain many somatic mutations [32] were amplified with mitochondrial-specific primers to avoid amplification of homologeous regions in the nuclear DNA. Five sets of mitochondrial specific primer pairs were used, resulting in amplification product between 714 and 928 base pair in length (see Table 1).

**Table 1:** Primers used to selectively amplify mtDNA

						Annealing
#	Start (b p)	End (b p)	Length (b p)	“Forward” primer (5′–3′)	“Reverse” primer (5′–3′)	Temperature (°C)
23	15924	201	846	^a^AACCGGAGACGAAAACCTTTTTC	^a^CTTTAGTAGGTATGTTCGCCTGT	51
1	16521	880	928	^a^CCATAAAGCCTAAATAGCCCACA	^a^CCAACCCTGGGGTTAGTATAGCT	54
10	6917	7671	754	^a^TGCTCTGAGCCCTAGGATTCATC	^a^TGAGGGCGTGATCATGAAAGGTG	55.5
16	10852	11566	714	^a^GCCTAATTATTAGCATCATCCCC	^a^ATGCCTCATAGGGATAGTACAAG	51
22	15169	15993	824	^a^GAGGGGCCACAGTAATTACAAAC	^a^TGGGTGCTAATGGTGGAGTTAAA	51

^a^ = tail sequence (CGCCCGCCGCGCCCCGCG)

The PCR reaction mixture contained 0.1 μl of extracted DNA (~5 ng), 0.8 mM dNTPs (0.2 mM of each dNTP) (VWR, Oslo, Norway), 1X Thermopol Buffer, 2 mM MgSO_4_, 0.075unit Taq/μl, 0.15 μM of each forward, reverse and fluorescently labeled primer (Integrated DNA Technologies, Leuven, Belgium) and total reaction volume of 10 μl. The temperature cycling was performed in an Eppendorf Mastercycler ep gradient S (Eppendorf, Hamburg, Germany) with an initial denaturation 94 °C for 240 s followed by cycling 38 times under the following conditions, denaturation at 94 °C for 15 s, annealing for 40 s with temperature given in Table 1 and elongation at 72 °C for 150 s.

### Capillary electrophoresis

All first round amplification products were checked and visualized by capillary electrophoresis in MegaBACE 1000 DNA Analysis System (GE Healthcare Life Sciences, Pittsburgh, PA, USA). Samples were loaded into the capillaries from 96-well plates by electrokinetic injection at 161 V/cm for 20 s. The temperature of the capillary chamber was set to 27 °C and electrophoresis was carried out at a constant field of 145 V/cm.

### Second round PCR

Template for second round PCR was 0.8 μl of a 1:200 dilution (first round PCR in H_2_O). The templates were dispensed into 96-wells plates with a syringe dispenser (Hydra 96, Robbins Scientific, USA). To each well 10 μl reaction mixture was added, consisting of 1xThermopol Reaction Buffer with 2 mM MgS04, 0.3 μM forward primer, 0.15 μM 1/2GC-tailed “reverse” primer, 0.15 μM, 6-Carboxyfluorescein-GC-clamp primer, 500 μM dNTP, 100 μg Bovine Serum Albumine (Sigma-Aldrich, Oslo, Norway) and 0.75U Cloned Pfu DNA polymeraze. Plates were sealed with two strips of electrical tape (Clas Ohlson, Oslo, Norway). The temperature cycling was repeated 25 times; 94 °C for 15 s, annealing temperature (given in Table 2) held for 30 s and extension at 72 °C for 60 s.

**Table 2:** Primers used to amplify fragments suitable for detecting mutation by CTCE

			Template,fragment#			Annealing
#	Start(bp)	End(bp)	fromfirstPCR	“Forward” primer(5′–3′)	“Reverse” primer(5′–3′)	Temperature (°C)
1	16569	25	1	^a^TGCATGGAGAGCTCCCGTGAGTGG	CCCCTTAAATAAGACATCACGAT	52
2	42	126	1	^a^ATTAACCACTCACGGGAGCTCTC	AGGATGAGGCAGGAATCAAAGAC	55
4	131	181	1	^a^CACCCTATGTCGCAGTATCTGTC	CACACTTTAGTAAGTATGTTCGC	55
6	483	153	1	^a^GGGGTTAGCAGCGGTGTGTGTGTG	TCCCACTCCCATACTACTAATCT	55
7	530	633	1	^a^TACCCAGCACACACACACCGCTG	CAAACCTATTTGTTTATGGGGTGA	55
8	673	705	1	^a^TTAGAGGGTGAACTCACTGGAAC	GGTTTGGTCCTAGCCTTTCTATT	58
82	7031	1734	10	^a^ACGACACGTACTACGTTGTAGCC	AATATGATAGTGAAATGGATTTT	52
84	7340	7416	10	^a^CTTTCTTCCCACAACACTTTCTC	TCTCAAATCATGAAAATTATTAAT	55
125	11029	11086	16	^a^TTAGGAGGGGGGTTGTTAGGGGGT	CATCCCTCTACTATTTTTTAACC	58
127	11193	11243	16	^a^ACCAGCCAGAACGCCTGAACGCA	GGTGTTGTGAGTGTAAATTAGTG	55
128	11283	11311	16	^a^TGTGCCTGCGTTCAGGCGTTCTGG	TAATCATATTTTATATCTTCTTC	60
130	11437	11492	16	^a^TTGACCCAGCGATGGGGGCTTCGA	GAGCCAACAACTTAATATGACTA	55
176	15201	15257	22	^a^AGAATCGTGTGAGGGTGGGACTGT	AGTAATTACAAACTTACTATCCG	60
177	15274	15377	22	^a^AGTAGACAGTCCCACCCTCACAC	GGTGATTTTATCGGAATGGGAGG	60
178	15394	15448	22	^a^CTAGGAATCACCTCCCATTCCGA	TAATGTCATTAAGGAGAGAAGGAA	55
181	15761	15864	22	^a^ACCTCCTCATTCTAACCTGAATC	CAGGCCCATTTGAGTATTTTGTTT	55
184	16080	16130	23	^a^CAAGTATTGACTCACCCATCAAC	ACAGGTGGTCAAGTATTTATGGTA	57
185	16170	16272	23	^a^GTGGGTGAGGGGTGGCTTTGGAGT	CCAATCCACATCAAAACCCCCTC	56
187	16263	16366	23	^a^AACTGCAACTCCAAAGCCACCCC	CCCTATCTGAGGGGGGTCATCCAT	58

^a^=tailsequence(CCCGCCGCCCCCGCCCGGG)

GC-Clamp=(6FAM-CGCCCGCCGCGCCCCGCGCCCGTCCCGCCGCCCCCGCCCGGG)

### Tumor analysis

Testicular tumor samples were obtained from six patients. Apparent normal tissues adjacent to the tumor were marked with a suture (by the surgeon responsible for removing the tumor) and snap frozen in liquid nitrogen. The suture was used as a reference when the sample was mounted in the cryotome. DNA extracted from the testicular tumor samples were analyzed for somatic mutations in the hotspot regions of the mtDNA. The fragments selected were based on the results of scanning 76% of the mtDNA in 94 tumor samples from 13 different tissues origin [32].

### Tissue sectioning

Sample with somatic mitochondrial mutations was mounted to a cryostat sample holder with a water-soluble glycols and resins matrix (Tissue-Tek® O.C.T. Compound, Sakura, Finetek, USA). The sample holder temperature was set to −20 °C and the knife temperature to −23 °C. The cryostat (Leica CM1950) cut 12 μm sections. Each cut was gently transferred to either a steel framed polyethylene naphthalate membrane (Leica, Leica Microsystems, Wetzlar, Germany) or glass microscope slide (Thermo Scientific, Gerhard Menzel, Braunschweig, Germany).

### Cycling temperature capillary electrophoresis

CTCE analysis was performed for the selected fragments as previously described [28]. In brief; a 96-capillary DNA analyzer (MegaBACE 1000) was used to analyze 6-carboxyfluorescein labeled PCR products. Mutant PCR amplicons were separated from the wild type by cycling the temperature around the capillaries. The cycling temperature was based on the theoretical melting temperature, for each fragment, calculated by Poland’s algorithm [29, 33] in the implementation described by Steger [34, 35]. The separation temperature proposed by the algorithms was adjusted based on the urea concentration in the matrix. The instrument was modified to allow for elevated temperature cycling [27, 36]. Temperature cycling was programmed in the macro.ini file used by the Instrument Control Manager (ICM) software package (GE Healthcare Life Sciences, Pittsburgh, PA, USA). The injection and running electric fields were as given for the first round amplicons.

### Internal standard

The two heteroplasmic mutations found in the initial sample analysis were re-amplified with a 5′ ROX-labeled primer. These were used as internal standards during electrophoresis and were injected into all capillaries in all runs prior to sample injection. The internal standard serves as a control of the separating temperature and as a marker for the DNA mutations.

### Tissue sectioning

#### Fixing and staining

The membrane and the glass slide with the 12 μm tissue section were dried at room temperature and fixed in pure methanol (Sigma-Aldrich) for 10 min, followed by subsequently air drying of the methanol residue. The tissue was stained with Giemsa azur eosin methylene blue solution (Merck, Damstadt, Germany) (diluted 1/20 with H2O) for 30 min. Slides are then submerged into 1% acetic acid in H2O solution for 30 s (differentiation) and immediately rinsed in water. The stained tissue was air dried prior to imaging by microscope.

### Laser capture microdissection

A Leica LDM 6000 was used to take images of tissue sections mounted on membrane or glass slides. The software, Leica laser microdissection V6.7.1.3952, was used to control the microscope when taking pictures or selecting areas for laser capture microdissection and cutting. A hardware modification was made to the collection unit allowing for samples to be collected into two strips of 8 PCR caps (VWR, Oslo, Norway).

20 μl of a collection solution (1×Thermopol buffer with Proteinase K, 0.27 μg/μl) was added to each cap in the inverted strips. After cutting and collecting the selected areas by laser capture microdissection, the strips (with collection liquid and tissue) were mounted onto a 96-well PCR plate (Axygen, VWR, Oslo, Norway). The plate was briefly centrifuged and incubated at 56 °C for 30 min. Deactivation of proteinase K was achieved by raising the temperature to 95 °C for 1 min. One microliter of incubated solution was used as template for the first round PCR (see above).

## Results

**Fig. 1:**
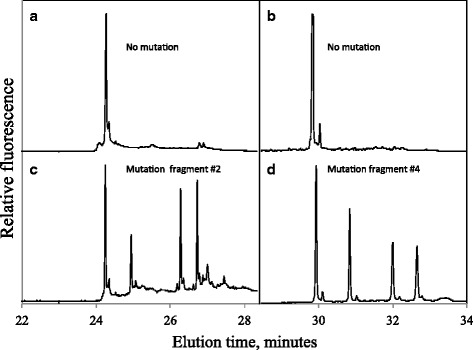
Electropherogram of mutated fragments detected by cycling temperature capillary electrophoresis. Electropherograms **a**) and **c**) show non-mutated samples, while electropherograms **b**) and **d**) show the two mutations found in one sample

Of the six testicular tumors analyzed, the sample from the Leydig cell tumor was found to have two mutations, one in fragment 2 and one in fragment 4 (Table 2). The two mutations were identified as base pair substitutions, C to T at base pair 64 and a T to C at base pair 152 (NC_012920.1). None of the other five tumors had any mutations in the fragments analyzed. Figure 1 shows the electropherograms for the fragments with a mutation. Finding only one sample with a mitochondrial mutation was less than expected, but is not significant when considering the small number of samples.

In the first step the DNA was extracted by needle biopsy taken through the tumor sample. It, therefore, contains a mixture of DNA from cells across the tumor. The presence of a detectable mutation fraction found in this way suggests that sampling by LCM might identify the same mutation. In the other tumors, no mutations were found, which suggests that none is present or the fraction is too low.

**Fig. 2:**
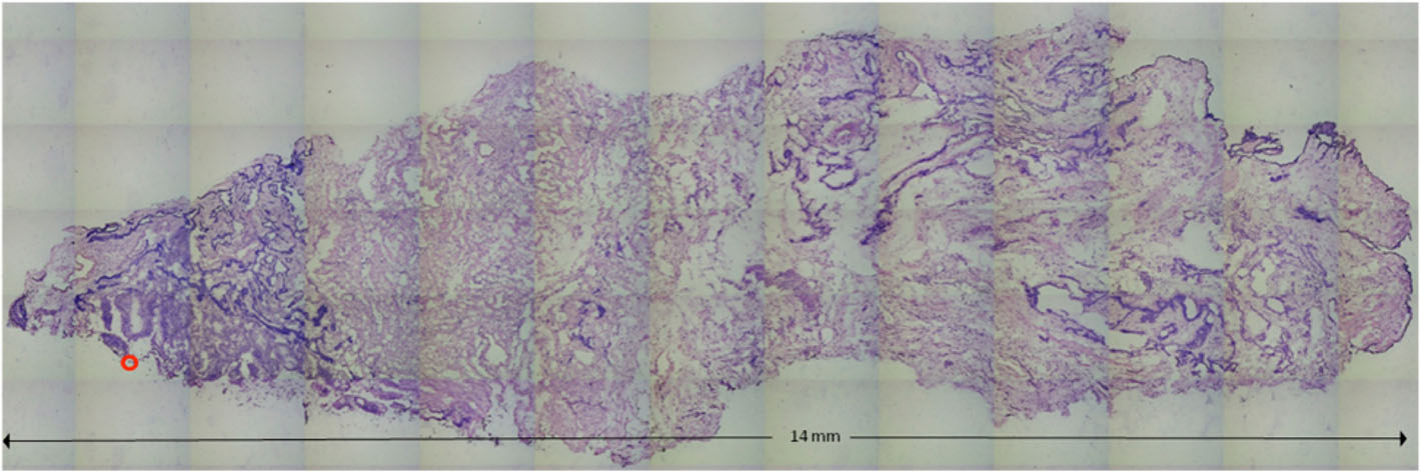
Overview of sample harboring two mutations. The *red circle* indicates suture position attached by the surgeon to indicate normal tissue. Image was taken with the LCM microscope by scanning the sample

The sample with two mutations was taken to the next step in the procedure. Figure 2 shows an image of a section of the tissue with two mutations. The red circle indicates the area determined as normal by the surgeon. Ninety-three circular spots (~25000 μm^2^) were dissected by LCM and dropped into separate caps. For each sample, fragments 2 and 4, in which a mutation was found, were analyzed.

To estimate the number of cells in a LCM sample, Giemsa stained dark blue nuclei were counted in 48 circles, by two researchers. The average number of nuclei observed was 31 with a standard deviation of 13 and a range of 7 to 76 nuclei/circle. The theoretical upper bound can be calculated assuming an average cell diameter of 20 μm. This gives a total of ~80 cells.

The mitochondrial DNA copy number in the LCM samples was determined by real-time PCR. LCM samples homoplasmic for both mutations (black circles) and wild type (white circles) were analyzed, and no difference in mtDNA copy number could be detected. The copy number was 300 per cell.

**Fig. 3:**
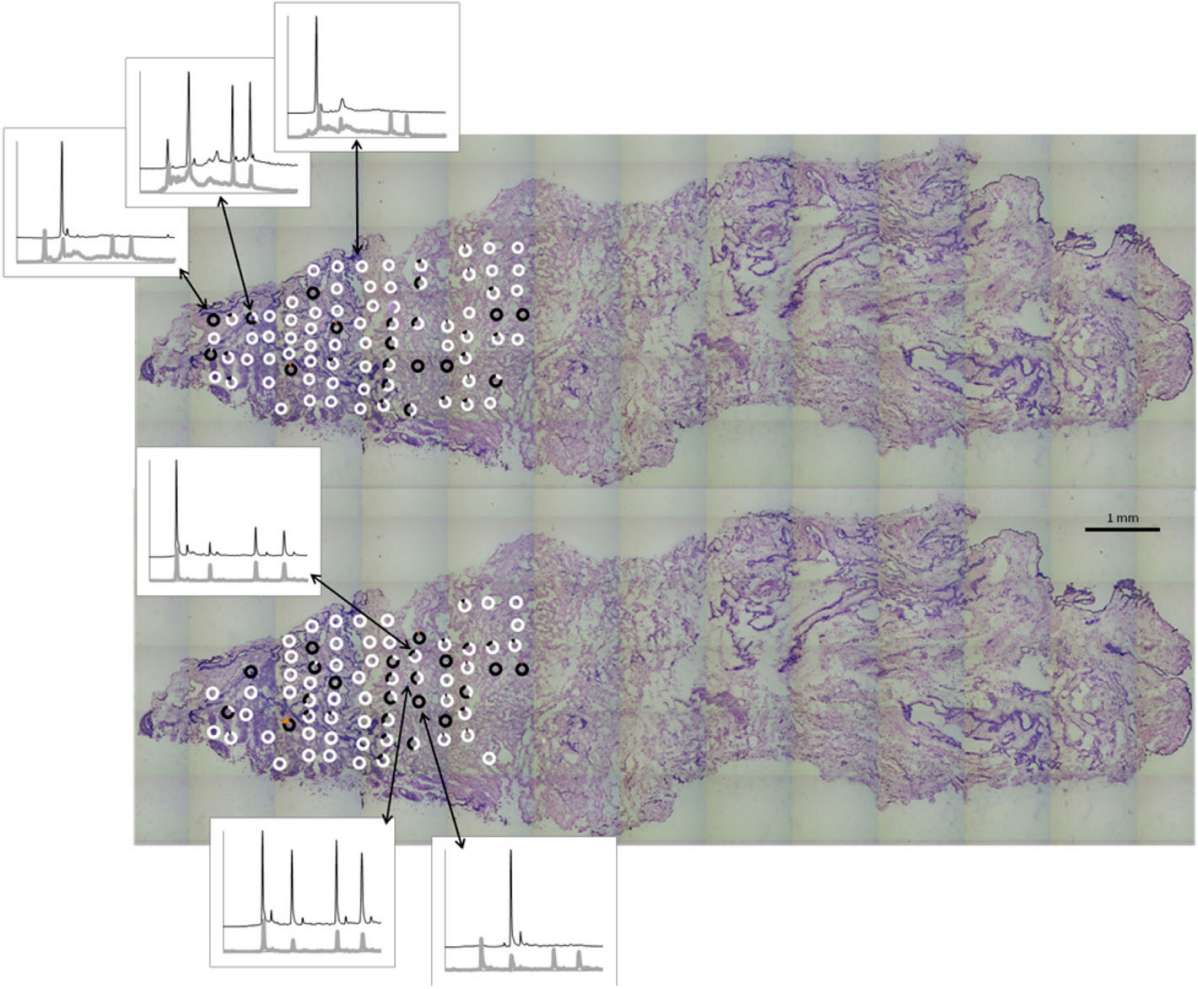
Laser capture microdissected spots with mutants fractions. Marker detected by primer set # 2 is on the top and marker detected by primer set #4 is on the bottom. *White* is associated with the first peak (homoduplex) in electropherogram, and *black* is associated with the second peak (homoduplex). The fraction of *white* and *black* in each circle, represent the fraction of the first and second variant respectively in each sample. Other color presents mutants not aligning with the internal standard in the electropherogram. Inserted electropherograms represent homoplasmic regions and heteroplasmic areas (e.g., mix of two cell population)

Figure 3 displays the LCM spots for which the mutant fraction could be determined for both mutations (color-coded by white and black circles). The combined LCM and PCR success rate was 86 and 76% for the two markers, respectively. In some samples, other mutants that do not co-elute with the internal standard have been marked with a different color. The inserted electropherograms demonstrate the allele separation and quantification of mutant fraction found in three different spots for each marker. The mutant fraction was calculated based on the peak heights and have previously been shown to have a linear relationship between signal and mutant fraction [29]. From these data, close to homoplasmic mutated regions could be identified for each marker.

**Fig. 4:**
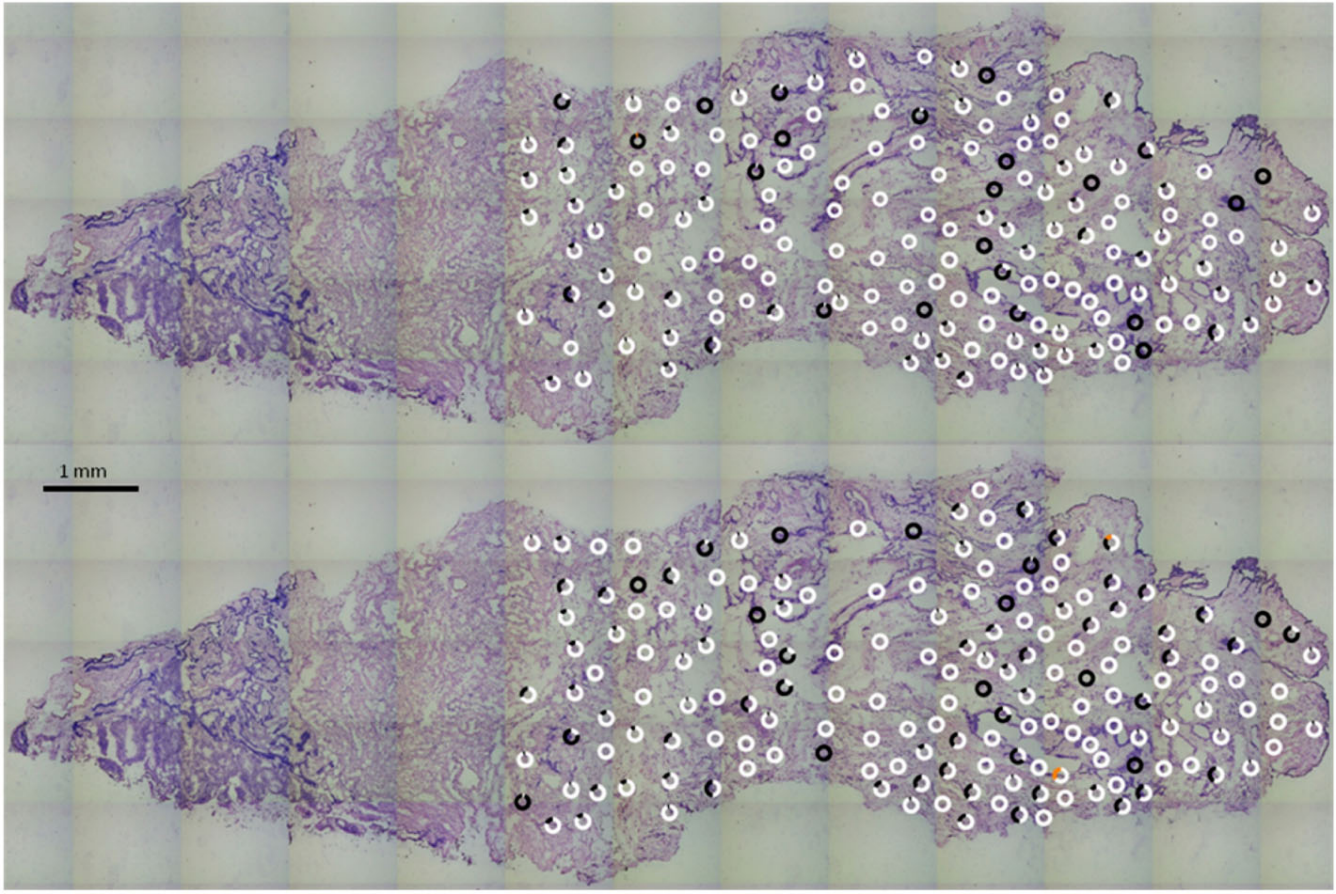
Laser capture microdissected spots with mutants fractions. Marker detected by primer set # 2 is on the top and marker detected by primer set #4 is on the bottom. *White* is associated with the first peak (homoduplex) in electropherogram, and *black* is associated with the second peak (homoduplex). The fraction of *white* and *black* in each circle, represent the fraction of the first and second variant respectively in each sample. Other color presents mutants not aligning with the internal standard in the electropherogram. Inserted electropherograms represent homoplasmic regions and heteroplasmic areas (e.g., mix of two cell population)

Following this first analysis, a further 186 LCM samples were taken from the remaining part of the tissue section, as shown in Fig. 4. The mutant fraction could be determined in a large fraction of samples (89% and 91%) as shown in Fig. 4. The location of the spots for the second part of the analysis were selected by a subjective assessment of visible structures in Fig. 4. Subsequent review of the data in Figs. 3 and 4 did, however, not reveal any clear relation between apparent tissue structure and observed mutant fraction.

**Fig. 5:**
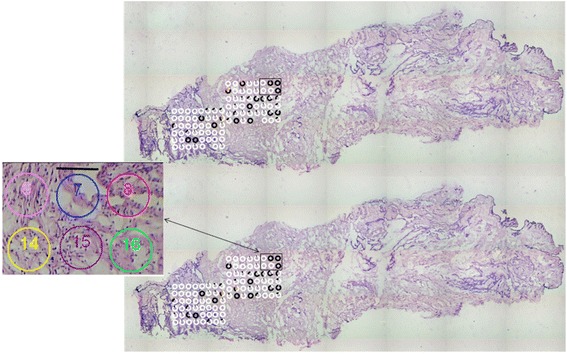
Focused laser capture microdissection in a grid of 8×6 spots. Marker detected by primer set # 2 is on the top and marker detected by primer set #4 is on the bottom. Enlargement show adjacent different homoplasmic areas. Scale bar is 178 μm long

To validate the procedure, a new section approximately 50 μm below the first one was analyzed. To eliminate possible biases, the samples were collected as two fixed grids (8x6) in Fig. 5. The success rate in determining the mutant fraction was 98 and 100%. The positions at which homoplasmic mutations can be observed in Fig. 5, correlate with those in Fig. 3. Six areas with near homoplasmic mutations in Fig. 3, are matched to six nearby areas in Fig. 5.

In addition, spots mostly show similar mutant fractions for both markers. The correlation was 0.75. It is reasonable to conclude that these mutations occur together, i.e., an mtDNA either contains both mutations or none.

**Fig. 6:**
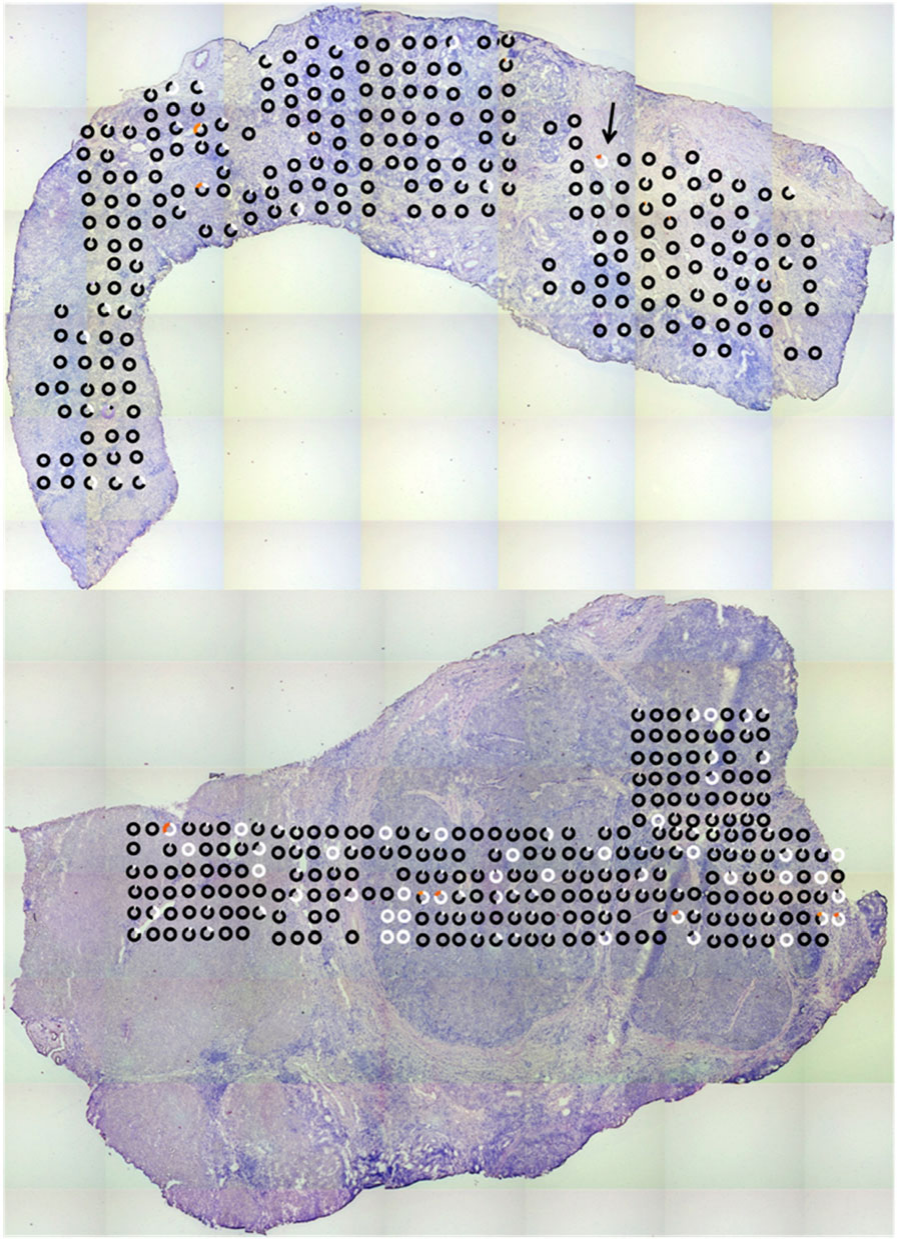
Laser capture microdissection of two liver samples. Top image is a cholangiocellular carcinoma, the lower is a hepatocellular carcinoma. The first peak in the electropherogram (homoduplex) is presented as *white* while second peak (homoduplex) is *black*. Other colors present represent mutants not aligning with the internal standard in the electropherogram (i.e., other variants)

The method was subsequently used to analyze two independent liver samples (a cholangio- and a hepato- cellular carcinoma). Both tumors had known mitochondrial mutations (study in progress). Each sample was subjected to LCM and mutation analysis in the grid–like sampling scheme as shown for the testis sample. Figure 6 displays the results of the 288 spots cut from each tissue. The upper section (cholangio carcinoma) is dominated by one mtDNA type and has heteroplasmic spots with a low mutant fraction in some areas. Only one spot, indicated by the arrow, is almost homoplasmic for the mutation, but in fact, the electropherogram showed the presence of a third mutation at a low fraction. The lower section of Fig. 6 (hepatocellular carcinoma) has multiple spots of near homoplasmic mutations as well as several heteroplasmic spots.

## Discussion

This paper describes a method to sample and analyze mutations from spots of a tumor tissue. The novelty lies in the small size of the samples or spots (25000 μm^2^ or an average of 31 cell) and the systematic distribution of the samples. Since each cell carries multiple mitochondria and each mitochondrion has multiple copies of mtDNA, dissecting such a small number of cells gives enough templates for PCR amplification. This would not be sufficient for detecting mutations in nuclear DNA. In our studies we observed that it is better to distribute the spots on a regular grid, rather than placing them according to visible structures. The results give a demonstration of the efficacy of tracing the distribution of mutant mitochondrial genomes across a slice from a tissue or a tumor. This method could be used for studies of humans or experimental animals.

Because the LCM instrument depended on gravitation, spots smaller than 25000 μm^2^ resulted in a higher laser capture failure rate (data not shown). Increasing the size of the spots risks dilution of the mutant fraction, leading to a potential sampling bias.

Because the probability of independent occurrence of an identical mutation in the mtDNA of a cell is very small, the observed results are consistent with a clonal expansion of cells. To place an upper bound on this probability, a fragment not having any mutation but situated in a hotspot region of the mtDNA (d-loop) was analyzed in 700 LCM samples. No mutations were found. The probability of independently acquiring a given mutation can thus safely be placed below 1/700. Some estimates put it much lower at around 10^−30^ [19]. To further strengthen the argument, macroscopic samples of 94 tumors were analyzed [32]. One hundred and forty-two high fraction mtDNA mutations were sequenced and none of them were identical.

In the specific case of the testicular tumor analyzed here, two homoplasmic mutations 88 base pairs apart are detected and traced. Since the presence of these mutations is highly correlated, it suggests that one cell lineage is being observed, and not a series of independent events.

The method described is sensitive and provides a quantitative way to explore any tissue for heteroplasmy. Under our optimized conditions, the cost of analyzing 300 spots is about $30 (not including the cost of a trained technician working for three days). Once heteroplasmy with a sufficiently large mutant fraction has been established, it can, for example, be used to explore the resection margins of a tumor. Furthermore, if a continuous stack of sections cut by microtome were available, investigating mitochondrial mutations in three dimensions becomes possible. This would advance the field of three-dimensional lineage tracing in humans.

## Conclusion

The method presented in this study can be used for lineage tracing in humans. Its capacity was demonstrated in human tumors and can be extended to the study of metastasis and normal tissue. Using a regular sampling grid of LCM spots is shown to be essential to study tumor heterogeneity.

## Abbreviations

CE: Capillary electrophoresis
CTCE: Cycling temperature capillary electrophoresis
LCM: Laser capture microdissection
